# Round about the Asylums.—X

**Published:** 1888-07-07

**Authors:** 


### Round About the Asylums.—X.

By a Medical Superintendent.

### LUNATIC HOSPITALS, PRIVATE AND PAUPER ASYLUMS.

In your correspondent's generally kind and complimentary notice of this institution in last week's Hospital there are several questions which I should be glad to answer, if you can favour me with the necessary space. He asks if registered hospitals such as this are to be blamed for not being wholly charitable hospitals, and for not opening their doors to those who have seen better days? and ought they to draw patients from private asylums? One reply only is possible to these questions under the circumstances. They cannot be wholly charitable in the sense in which he puts it, because most of them have no endowments whatever, and have an income from which to dispense charity only from the funds which are derived from a fair proportion of patients whose payments exceed their actual cost. From this source they contribute largely in aid of the payments of those who have seen better days and are now poor, and they derive patients from the class which provides the inmates of private asylums, because all the surplus which thus accrues is known to be so applied, after every requirement of the paying class has been met on a liberal scale. The public recognize the non-proprietary element, and favour it. A table showing the actual payments after reduction, would prove nothing, because it would be easy to reduce the comforts and conveniences of the patients *pari passu* with their payments. That the cost of maintenance is large, and that ten patients are maintained for nothing, and a large multiple of that number for less than they cost, proves conclusively that much charity is done, even if it were not stated that £2,500 had been so expended during the year. The low rate of 1·9 for deaths was, of course, exceptionally small, but your correspondent must have seen that the average of the last ten years was 4·7 per cent. Paralytics, epileptics, and such cases are not excluded. The patients are taken as application is made, when there are vacancies, and unless under very exceptional circumstances none are refused because of any supposed unsuitableness in consequence of their mental condition.

Fred. Needham, M.D.

Barnwood House, Gloucester.

[While inserting Dr. Needham's letter, we repeat our dislike to making the columns of The Hospital a medium of controversy on the subject. It will be inferred, however, that our answers to the question would hardly be the same as Dr. Needham's. It is beyond dispute that county asylums contain widows and children of clergymen, medical men, etc., and that the so-called charitable hospitals are receiving patients at £4, £5, or perhaps even £10 a week; each one of these occupying room enough for two or three "poor" cases. We cannot help thinking that a table such as we suggested would teach us much. Where is the charity in receiving a patient at 30*s*. or 40*s*. a week when accommodation can be obtained for similar sums in private asylums? Whether the latter ought to exist is an open question; but does it mend matters to take paying cases from these institutions? Surely a Gloucester tradesman would have good reason to complain if the Charity Commissioners subsidized another tradesman in the same line. We are pleased to learn that epileptics and paralytics are received at Barnwood House, and we hope the next annual report will show the numbers under treatment during the year.]

### SUSSEX COUNTY ASYLUM.

In the resignation of Dr. Williams, after a service of seventeen years as medical superintendent, the asylum has sustained a great loss, and one from which it will not easily recover. The visitors unanimously recommended a pension of £500 a-year, which was at once confirmed by Quarter Sessions, and while congratulating Dr. Williams on the result, we regret that ill health was the cause of his resignation. The visitors state that Dr. Williams' health broke "down under the strain of an unceasing devotion to the duties of his office," and "that he rendered highly valuable services to the ratepayers of the county." Everyone will agree with this opinion. There are 833 patients in the asylum, showing an increase of 9 in the numbers for the previous year; 228 patients were admitted, 122 were discharged, and 97 died. The recoveries were 39·4 per cent. on the admissions, and the deaths were 10·6 per cent. on the average number resident. Looking into the causes of insanity in those admitted, we find that hereditary influence heads the list, being the assigned cause in 38 of the cases; domestic trouble, adverse circumstances, and mental anxiety collectively account for 37; drink for 26; love for 10; and religion for 5. Early treatment is once more insisted on as essential in insanity. Dr. Williams says: "It should be treated as soon as possible, and lunatics, especially acute cases, should be sent to the asylum at once, and not detained in workhouses or at their homes in the hope of the attack passing off." Scarlet fever attacked four patients and one attendant. The detached hospital was brought into use, and the disease did not spread further. The weekly cost of maintenance was 8*s*. 9*d*.; out-county patients are charged 14*s*., and private cases 16*s*. a-week. There are 15 of the latter class. The Commissioners say: "Though we arrived at the asylum before 10 a.m., we found all the day-rooms in good order, bright, and comfortable, the patients properly dressed, and the condition of the wards and the patients creditable to the superintendent and his staff."

### NORTHAMPTON COUNTY ASYLUM.

Here the system pursued is exactly opposite to that noticed when speaking of Denbigh, Lincoln, and some others. The Northamptonshire visitors have always anticipated the wants of their county, and the result of this policy is shown by the following extract from the report of the committee: "The total number of patients in the asylum on December 31st was 704; 498 of these are chargeable to the county of Northampton and its boroughs. The out-county and private patients have increased by 16, and now number 206." The rate for in-county patients is 7*s*. 6*d*., for out-county 14*s*., and the private patients pay 17*s*. 6d. and under. A new block is being built for the separate treatment of idiot children, an object which certainly deserves success. The recoveries were 29·7 per cent. on the admissions, and the deaths were 12·3 per cent. on the average number resident. We learn that the open-door system has been tried here, and apparently with success. The superintendent states in his report that "the system was begun very cautiously in this asylum three years ago, and since then it has been gradually extended, until at the present time all the day-rooms, except one on each side, are left unlocked by day. The escapes were fewer than in former years. Probably this is a mere coincidence; but certainly no inconvenience has resulted from the unlocked doors. There is always something prisonlike in the grating noise of a key in a lock, and to banish anything prisonlike from an asylum must ever be a change for the better if it can be carried out with safety." The jubilee was celebrated on June 21st, and we are told that 450 patients joined in the games and sports, and that a small present was given to each. The Commissioners say that "the condition of this asylum continues to be satisfactory."

